# Vitamin A deficiency alters the pulmonary parenchymal elastic modulus and elastic fiber concentration in rats

**DOI:** 10.1186/1465-9921-6-77

**Published:** 2005-07-20

**Authors:** Stephen E McGowan, Erika J Takle, Amey J Holmes

**Affiliations:** 1 Department of Veterans Affairs Research Service and Department of Internal Medicine, Roy A. and Lucille J Carver College of Medicine, University of Iowa, Iowa City, IA, USA

**Keywords:** Elastin, retinoic acid, emphysema, bronchial hyperreactivity, cholinergic

## Abstract

**Background:**

Bronchial hyperreactivity is influenced by properties of the conducting airways and the surrounding pulmonary parenchyma, which is tethered to the conducting airways. Vitamin A deficiency (VAD) is associated with an increase in airway hyperreactivity in rats and a decrease in the volume density of alveoli and alveolar ducts. To better define the effects of VAD on the mechanical properties of the pulmonary parenchyma, we have studied the elastic modulus, elastic fibers and elastin gene-expression in rats with VAD, which were supplemented with retinoic acid (RA) or remained unsupplemented.

**Methods:**

Parenchymal mechanics were assessed before and after the administration of carbamylcholine (CCh) by determining the bulk and shear moduli of lungs that that had been removed from rats which were vitamin A deficient or received a control diet. Elastin mRNA and insoluble elastin were quantified and elastic fibers were enumerated using morphometric methods. Additional morphometric studies were performed to assess airway contraction and alveolar distortion.

**Results:**

VAD produced an approximately 2-fold augmentation in the CCh-mediated increase of the bulk modulus and a significant dampening of the increase in shear modulus after CCh, compared to vitamin A sufficient (VAS) rats. RA-supplementation for up to 21 days did not reverse the effects of VAD on the elastic modulus. VAD was also associated with a decrease in the concentration of parenchymal elastic fibers, which was restored and was accompanied by an increase in tropoelastin mRNA after 12 days of RA-treatment. Lung elastin, which was resistant to 0.1 N NaOH at 98°, decreased in VAD and was not restored after 21 days of RA-treatment.

**Conclusion:**

Alterations in parenchymal mechanics and structure contribute to bronchial hyperreactivity in VAD but they are not reversed by RA-treatment, in contrast to the VAD-related alterations in the airways.

## Background

Previous studies have shown that vitamin A deficiency (VAD) in rats is associated with a decrease in gas-exchange surface area, a decrease in the bronchial elastic fiber density, and with an increase in airway responsiveness to cholinergic agents [1,2]. Although VAD is uncommon in economically developed countries, it remains an important public health problem in the developing world particularly in children during the first seven years of life, when pulmonary alveolarization occurs [3]. Vitamin A and its active metabolite retinoic acid influence alveolar development and restoration, however the mechanisms responsible for these effects remain poorly understood [4,5]. In our experimental model of VAD, rats do not become deficient until after the period of maximal alveolar formation, which is completed by 3 weeks of age [2,6]. During these first 3 weeks of postnatal life there is an increase in the mRNA for tropoelastin, the soluble precursor of cross-linked elastin, which is an important determinant of the mechanical properties of the lung parenchyma and airways [7]. Once it is cross-linked, elastin normally undergoes very little turnover, although this does occur in pathological conditions such as emphysema [6,8].

In order to better identify the mechanisms that are responsible for airway hyperreactivity in VAD rats, with respect to morphological and biochemical characteristics of the pulmonary elastic fiber network, we evaluated the mechanical properties of the lung parenchyma that are most involved in regulating small airway diameter. Airway responsiveness to cholinergic agents is influenced by airway-parenchymal interactions [9]. The elastic fibers in the walls of alveoli and alveolar ducts, which form a continuous network with elastic fibers in the small and larger airways, are an important structural determinant of these interactions [10,11]. The elastic fibers within the airway connect the epithelial basement membrane to the smooth muscle layer [11]. Fibers in the adventitia that surrounds the airway smooth muscle are connected to parenchymal elastic fibers located in the surrounding alveoli and alveolar ducts. The contractile cells in the alveolar ducts may also influence airway smooth muscle contraction because contractile cells in the two locations are connected through the intervening elastic fiber network [11]. Physiological measurements of the elastic modulus of the lung are sensitive to alterations in both the airways and the parenchyma [12]. For an isotropic material, the ability to resist volume and shape distortion, respectively, is described by the bulk modulus (*k*, which is proportional to the ability to resist uniform expansion) and the shear modulus (μ, which is proportional to the ability to resist a small isovolume shape distortion). The lung is more constrained in volume expansion than in shape distortion, and *k *increases exponentially with volume whereas μ increases arithmetically [13]. There are three mechanisms whereby the lung resists deformation: (a) altering the spacing between microstructural elements, (b) altering the orientation of the microstructural elements, and (c) stretching of the microstructural elements [12]. Any or all of these three factors may be affected if there are abnormalities of the elastic fiber network. In pulmonary emphysema there are changes in all three mechanisms. Dilated alveoli and alveolar ducts increase the spacing between elastic fibers, elastic fibers are disarrayed and are abnormally connected, and the remaining alveolar walls and ducts are stretched by dilation. The elastic modulus of the lung parenchyma may also be altered in VAD rats, which have fewer and dilated gas exchange units compared to the lungs of VAS rats [1]. Because the inhalation of aerosolized cholinergic agents distorts the lung parenchyma producing inter-dispersed regions of localized hyperinflation and atelectasis, one would predict that alterations in the elastic modulus would be accentuated after cholinergic administration [14]. We hypothesized that because of parenchymal distortion and localized hyperinflation, cholinergic administration would produce a larger increase in the bulk modulus of VAD compared to vitamin A sufficient (VAS) rat lungs. To address this hypothesis we have characterized the effects of VAD on parenchymal mechanics and elastic fiber architecture. We have studied elastic fiber length per unit volume of lung, elastin production, and measured the elastic modulus of the lung parenchyma in VAS and VAD rats before and after the administration of CCh. We further hypothesized that if the elastic fiber network was a major determinant of the bulk and shear moduli, then restoration of the elastic fiber network may restore the elastic moduli to values that are similar to those in VAS rats. Therefore, we administered retinoic acid (RA) to determine whether reversing the tissue effects of VAD would coordinately reverse abnormalities in the elastic fibers and in the bulk and shear moduli. The elastic fiber length per unit volume was decreased in VAD rat lungs and may have contributed to the observed differences in shear modulus. However, other architectural modifications accounted for the observed differences in the bulk modulus in VAD compared to VAS rats.

## Methods

### Production of Vitamin A Deficiency

Specific pathogen-free female Lewis rats were obtained from Harlan-Sprague Dawley (Madison, WI). All animals were maintained in HEPA-filtered cages and sentinel animals were used to establish that the colony remained specific-pathogen free. The protocol was approved by the animal use committees at the Veterans Affairs Medical Center and the University of Iowa. The rats were weaned at postnatal day 21 and placed on a VAD diet-modified (catalog number 96022, ICN Corp., Aurora, OH), for 7 to 10 weeks to achieve vitamin A deficiency [15]. Vitamin A sufficient rats were littermates of the VAD animals or age-matched females were purchased from Harlan-Sprague Dawley. The general health of the VAD rats was monitored and the VAD animals were used prior to the onset of weight loss or keratitis. We have previously shown that this protocol consistently produces vitamin A deficiency [1]. The onset of VAD was identified by the cessation of weight gain which occurred earlier than in females who were fed the control diet. When the VAD rats stopped gaining weight, they received 25 μg of retinyl acetate that was administered orally at weekly intervals to prevent weight loss and a generalized nutritional deficiency. Twenty-five micrograms of retinoic acid (RA), in safflower oil, were administered orally daily for 12 or 21 days to some rats to determine whether this reversed the effects of VAD. Supplementation of VAD rats with RA for 12 days is sufficient to completely restore the expression of retinaldehyde dehydrogenase, a retinoid responsive gene [16].

### Analysis of the elastic modulus of the distal lung

Rats were anesthetized, the trachea was cannulated with a 14 gauge catheter, and the animals inhaled 100% oxygen for 6 minutes. The tracheal cannula was plugged, a medium sternotomy and laparotomy were performed, and the heart was allowed to pump for 5 minutes to induce total pulmonary atelectasis. After exposing the heart, the lungs were perfused with 15 ml of 137 mM NaCl, 8 mM Na_2_HPO_4_, 2.7 mM KCl, 1.5 mM KH_2_PO_4_, pH 7.4 (PBS) to clear the pulmonary circulation. The trachea, mediastinum, heart, and diaphragm were excised en bloc and the preparation was immersed in PBS. The lungs were inflated with 0.2 ml of air every 5 s over approximately 3 minutes to a constant pressure of 25 cm and then allowed to collapse to 0 cm H_2_O pressure, and the inflation and deflation were repeated once. The deflation volume-pressure curve was assessed (method described subsequently) both before and after the intratracheal administration of 16 mg/ml of carbamylcholine (CCh) during ventilation of the lungs with a tidal volume of 0.3 ml for 90 s using a DeVilbiss AeroSonic ultrasonic nebulizer [1]. In each case, the lungs were inflated once to 25 cm H_2_O pressure and deflated to 0 cm prior to the inflation phase of the volume-pressure analysis.

Studies were performed to evaluate the elastance of the distal lung by ventilating the excised lungs at a small tidal volume (0.3 ml) with a volume-cycled rodent respirator (Inspra, Harvard Apparatus, Holliston, MA). Flow was measured by a pneumotachograph attached between the mechanical ventilator and the endotracheal tube, and volume was calculated by integrating the flow. Tracheal pressure was measured continuously and data were acquired and sampled at 50 Hz using a RSS 100HR Research Pneumotach system (Hans Rudolph, Kansas City, MO). Ventilating at 0.3 ml minimized minimized air-trapping. The resistance (R) and elastance (E) were calculated from the equation P_L _= R_L_Q + EV + K where K is a parameter reflecting the end-expiratory pressure, Q = flow, and V = volume [17].

We followed the methods that have been described by Salerno and Ludwig for evaluating the bulk modulus (*k*) and the shear modulus (μ) of rats [18]. Bulk modulus (*k*) is expressed by the equation *k *= V·dP/dV and changes with the absolute volume of the lung. The *k *was calculated from the incremental changes in P and V, over the 0.44 s that were required for the ventilator to deliver 0.3 ml, and expressed as the mean of 5 inflations. The shear modulus was calculated from the equation G/2wD = μ/[1-(3*k*-2μ)/2(3*k*+μ)] where G = the lung's resistive force against the displacement, w = the displacement of the punch, D = diameter of the punch, *k *= bulk modulus and μ = shear modulus [19]. The end tidal volumes of the preparations were controlled by adjusting the positive end expiratory pressure (PEEP) to 3 cm or 8 cm. The shear modulus was measured by the punch-indentation test (using a punch with a diameter of 0.45 cm and advancing it by 0.5 mm increments) at the same inflation volumes by adjusting the airway pressure to 3 or 8 cm using a biased flow of air, an adjustable valve and a pressure transducer [18]. The volume at atmospheric pressure was assessed by volume displacement [20]. The absolute volume of the lung at 3 cm H_2_O was calculated by adding the volume that yielded this pressure during the volume-pressure maneuver to the residual volume. The absolute volume at 3 cm H_2_O pressure did not change with the administration of CCh. The bulk modulus and shear modulus were analyzed at 3 cm and 8 cm H_2_O prior to administration of CCh and at 3 cm after CCh administration. All of the measurements were completed within 90 minutes after euthanizing the animals.

### Analysis of elastin

The right lungs of the rats that were used for the analyses of elastic moduli were frozen in liquid nitrogen, without separation of the bronchovascular bundles from the parenchyma. A portion of the lung was extracted with chloroform and methanol, dried under vacuum and weighed (the dry-defatted weight) [21]. The dried lung tissues were used to isolate elastin by extracting with 0.1 M NaOH at 98°[4]. The washed, alkali-resistant insoluble elastin residue was hydrolyzed for 20 hours in 6 N HCl under vacuum and the HCl was removed by evaporation under a stream of nitrogen. The amino acid composition of the hydrolysate was analyzed using reverse-phase HPLC following a procedure that has been described previously [4]. The elastin contents were normalized to the dry-defatted weight of the lungs.

### Analysis of the pressure-volume characteristics of VAS and VAD lungs

A deflation volume-pressure curve was generated for the excised lungs before and immediately after exposure to carbamylcholine. The lung was inflated to 25 cm H_2_O pressure over 90 s and deflated in 0.5 ml increments using a Harvard PHD 2000 programmable syringe pump, pausing for 12 seconds at each volume before recording the pressure. Pressure was measured using a Validyne Model DP45-28 (Validyne, Northridge, CA) pressure transducer. The signal was conditioned by a Validyne carrier-demodulator and sent to a strip-chart recorder. The transducer was calibrated using a water manometer. The volume-pressure data that were obtained at volumes from 80% to 30% total lung capacity were subjected to a double logarithmic transformation. Linear regression analysis was applied to the normalized data to calculate the slope of the deflation volume-pressure curve. [22].

The effects cholinergic administration on hysteresis in VAS and VAD rats was analyzed in a separate set of experiments. The thoracic cavity was entered by a median sternotomy and the chest wall was widely retracted. The abdominal contents were deflected with a retractor, the rats were euthanized by exsanguination, and the lungs were perfused with heparinized PBS. The lungs were inflated with 10 ml of air and allowed to return to residual volume, and the inflation and deflation were repeated once. Then the lungs were inflated in one ml increments up to 10 ml and then deflated in one ml increments, pausing for 12 seconds at the end or each increment prior to the pressure measurement. Methacholine (16 mg/ml) was delivered as an aerosol for 90 sec and the lungs were inflated once with 10 ml of air and allowed to return to residual volume. Then the incremental inflation-deflation maneuver was repeated to assess the effects of methacholine. The tracheal pressure was plotted at each increment and the hysteresis ratio was calculated using Microsoft Excel and a specially designed macro (Huvard Research and Consulting, Virginia Commonwealth University) [23].

### Elastic fiber concentration in respiratory airspaces

Left lungs were fixed at 20 cm H_2_O pressure for 16 hours at 4° in 4% paraformaldehyde and the volumes were determined by displacement [24]. The mean volumes of the left lungs did not vary significantly according to retinoid status and were 3.63 ± 0.18, 3.62 ± 0.12, and 3.83 ± 0.09 for VAS, VAD and VAD + 12d RA, respectively (n = 5 for each group). The fixed lungs were cut into sagittal slices of approximately 1.5 mm thickness. The slices were cut into strips of approximately 3 × 2 mm. The lungs were cut prior to dehydration, because it was difficult to uniformly dehydrate them. Therefore the displacement volumes were not measured after dehydration. The strips were then dehydrated in progressively increasing concentrations of ethanol (from 50 to 100%). The ethanol was replaced with 2 exchanges of LR-White resin, the strips were placed in gelatin capsules, and the LR-White was allowed to polymerize overnight at 60°. Sections were cut at a nominal thickness of 2 μm using a diamond-titanium knife and the actual thickness was determined using a stylus profilometer. Sections were hydrated, stained with orcein-hematoxylin, dehydrated and mounted in resinous medium. The intersections of alveolar septal elastic fibers with a test line were enumerated in 50 microscopic fields per section at 1000× magnification. The test line was a line spanning the width of a reticule placed in the ocular. The average number of intersections of a structure with a test line is one-half the ratio of the length to the volume [25]. Therefore the length of elastic fibers per unit volume (L_v_) is equal to 2 times (average number of intersections / length of test line) times the thickness of the section. This value for elastic fiber length per unit volume is a measure of elastic fiber concentration and will be referred to as "concentration" [25]. The gas-exchange (included both alveoli and alveolar ducts) surface area was determined using previously described methods [1]. Randomly chosen paraffin blocks of the left lung were sectioned and stained with hematoxylin and eosin. One section per rat was randomly selected and 6 fields per section were photographed at 50 × at random avoiding blood vessels and airways. The photographs were uniformly enlarged, overlaid with transparent grids and analyzed using morphometric methods [26]. The volume densities of airspace and tissue were determined by point counting using a 10 by 10 grid with 100 evenly spaced points, ~42 μm apart, as described previously [27]. Mean cord lengths (L_m_) were determined by counting intersections of airspace walls (including alveoli and alveolar ducts) with an array of 70 lines, each ~33 μm long [28]. The mean cord length is an estimate of the distance from one airspace wall to another airspace wall. The volume densities of the airspace and tissue, the mean cord length and the alveolar surface area were calculated as described previously [28]. Surface areas were expressed per cm^3 ^of distal lung tissue.

### Histological assessment of airway contraction

Approximately 20 minutes (the time required to measure the bulk and shear moduli) after administering the CCh (or in the absence of CCh-administration), the left lung was inflated to 16 cm H_2_O pressure with a stream of air, deflated to 5 cm, and then frozen in vapors of liquid nitrogen. The tissue was fixed by freeze substitution to maintain the architectural relationships that existed at the time of freezing. Carnoy's fixative (60% ethanol, 30% chloroform, and 10% acetic acid) was cooled with dry-ice and maintained overnight in a -20° freezer with excess dry-ice [14]. The following day, progressive concentrations of ethanol were substituted for the Carnoy's fixative while the lungs were maintained at -20° until 100% ethanol was reached [9]. The tissue was maintained in 100% ethanol overnight at -20° and then at 4° for 24 hours. The lungs were then embedded in paraffin, sectioned and stained with hematoxylin and eosin. Airways that contained a continuous circumference of smooth muscle and had been sectioned transversely were selected, photographed, and 35-mm slides were prepared. The 35 mm slides were digitized, the digitized images were analyzed using Image J (public domain software available at ), and the perimeter of the epithelial basement membrane, the lumen, and the inner and outer borders of the smooth muscle were traced. A stage micrometer was photographed at various magnifications and the micrometer-images were digitized using the same settings (scan resolution and enlargement) that were used for the airways. This allowed a conversion from pixels to microns. The actual area (A) of the airways that was luminal to the basement membrane was compared to the calculated area for the airway in the fully dilated (un-contracted) state (Ar). The details of the methods have been described and are predicated on the observation that the epithelial basement membrane circumference (perimeter) remains unchanged with constriction [29]. This allows one to relate all measurements to the ideally relaxed area that is contained within the circumference of the basement membrane, A_r _= BM^2^/4π. The A/A_r _is an index of the degree of airway narrowing and is influenced by both the fixation pressure and smooth muscle contraction [29,30]. Only airways with a ratio of the smallest to the largest diameter that was greater than 0.6 were used for the analysis of A/Ar. We stratified the A/Ar according to airway size because others have shown that airway diameter itself is a determinate of the contraction index [30].

### Physiological assessment of lung parenchymal distortion

Immediately prior to euthanasia four VAD and four VAS rats were exposed to an aerosol of CCh for 60 seconds, whereas three VAS and three VAD rats were not exposed to CCh. The lungs were quickly removed and the left lung was inflated at 10 cm H_2_O pressure and fixed by freeze substitution, as described previously. Ten cm of pressure was used instead of 5 cm, because the lower inflation pressure was insufficient to provide uniform expansion, and an initial inspection of lungs fixed at 5 cm H_2_O suggested that the mean chord length could not be accurately determined. Paraffin embedded lungs were sectioned, 9 randomly selected fields from each lung, which contained alveoli and alveolar ducts were photographed at 25× magnification, and digitized images were prepared as described previously. The images were uniformly enlarged, overlaid with an array of lines, and the Lm was determined as previously described. To evaluate the variability of airspace size, the standard deviation of the Lm (SD Lm) was assessed for each lung. The means of the SD Lm determinations for four CCh-exposed and three unexposed lungs VAS and VAD lungs were calculated. To assess the proportion of alveolar and alveolar duct walls (as opposed to airspace) in the sections from lungs fixed at 10 cm H_2_O, the digitized images were subjected to uniform thresholding to separate air and tissue densities. The number of pixels that corresponded to tissue density (termed the atelectasis index or ATI) was determined for each microscopic field (the same images that were used to determine Lm) [9]. The proportion of pixels corresponding to tissue density was expressed relative to the total number of pixels in the microscopic field, which was the same for all of the images. To assess variability of the tissue density, the standard deviation of the ATI (SD ATI) was assessed for each lung. The means of the SD ATI determinations for four CCh-exposed and three unexposed lungs VAS and VAD lungs were calculated.

### Statistics

The results were expressed as mean ± SEM and statistical comparisons were made using analysis of variance (ANOVA with a Student-Newman-Keuls post-hoc test). Differences were considered significant if p was less than 0.05. (n) is the number of animals in each treatment group, except for the morphometric studies in which (n) is the number of airways or lung parenchymal sections that were analyzed for each vitamin A-treatment group.

## Results

### VAD increases the elastance of excised lungs

**Figure 1:**
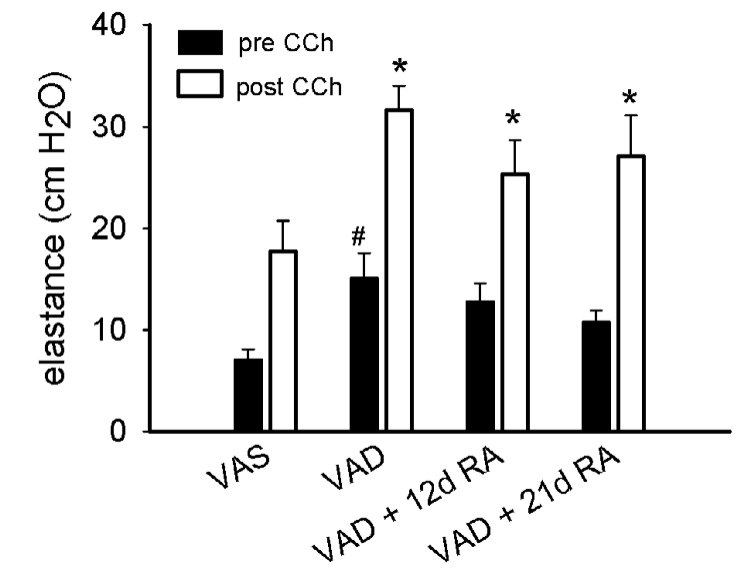
**Effects of vitamin A deficiency (VAD) on the elastance of excised lungs**. After standardizing the volume history by inflating to 25 cm H_2_O, the excised lungs were ventilated at a tidal volume of 0.3 ml and 3 cm of PEEP. Elastance (mean ± SEM, n = 7 in each group) was calculated prior to (solid bars) and after (open bars) administration of aerosolized carbamylcholine (CCh). (#) p < 0.05, VAD compared to vitamin A sufficient (VAS), prior to CCh. (*) p < 0.05, VAD, VAD + 12 days (d) and VAD + 21 d of retinoic acid (RA) compared to VAS, after CCh. 2-way ANOVA, Student-Newman-Keuls post-hoc test.

The vitamin A deficient diet led to a decrease in the hepatic retinyl ester contents from 768 ± 248 nmol/g in VAS rats to 17.5 ± 5.2 nmol/g and 14.5 ± 1.9 nmol/g in VAD rats that remained unsupplemented or were supplemented with RA for 12 days, respectively, consistent with a vitamin A deficient state. The elastance of excised lungs that were ventilated at a tidal volume of 0.3 ml and 3 cm PEEP was significantly higher in VAD than in VAS rats in the absence of CCh (Figure 1). Following the administration of CCh, the elastance increased in all three categories of retinoid status. And the CCh-related increase in elastance was significantly higher for VAD and VAD rats that had received RA for 12 days than for VAS rats. These findings were consistent with our previous findings for the lungs *in situ*, using larger tidal volumes, except that the 12 days of RA-treatment did not lower the elastance of the excised lung to a level that was similar to that for VAS rats [1]. We next determined the effects of CCh on the bulk and shear modulus components of the elastic modulus.

### VAD increases the elastic modulus after CCh-administration

**Figure 2:**
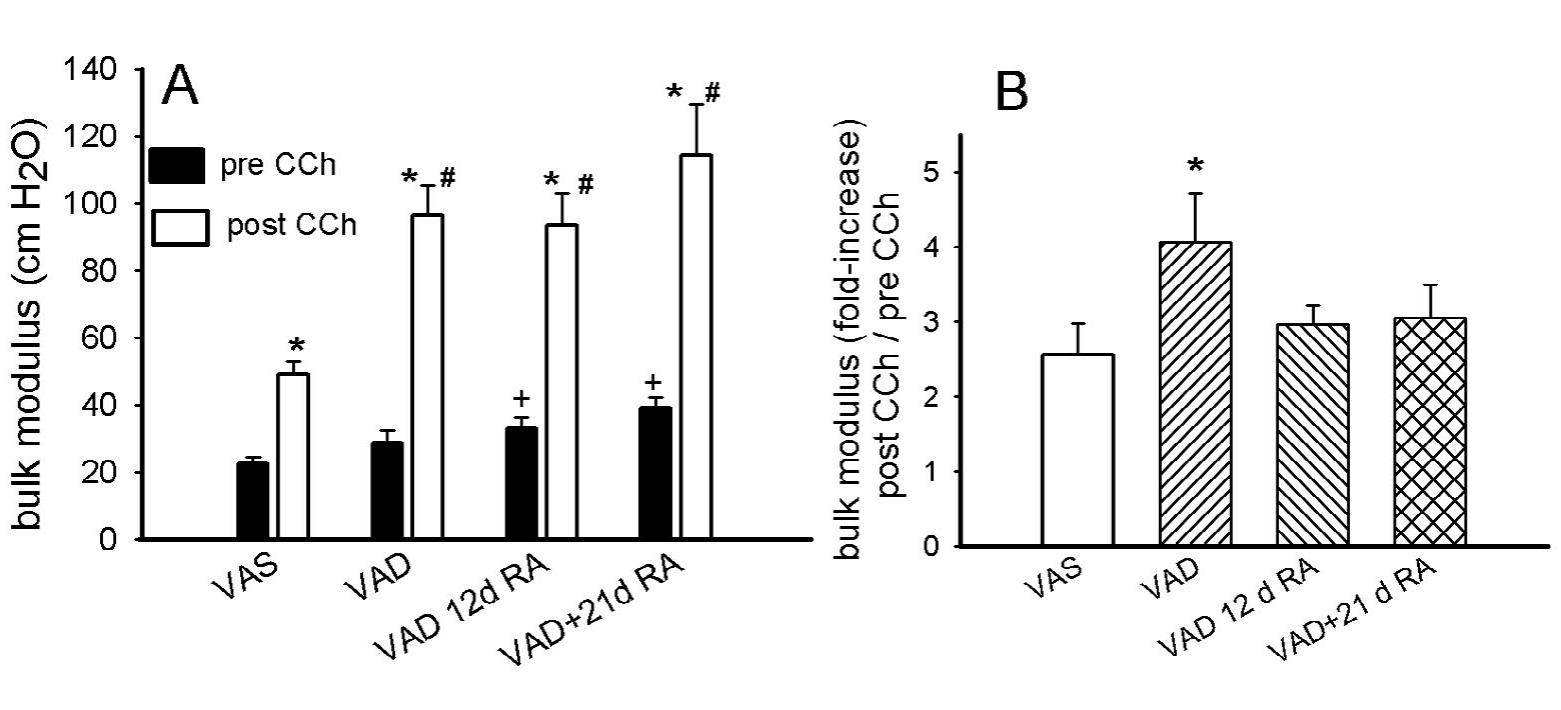
**Bulk modulus is increased in vitamin A deficient rats (VAD)**. **(A) **Bulk modulus (mean ± SEM, n = 9 in each group) was increased (*, p < 0.05) by carbamylcholine (CCh) administration (open bars) in vitamin A sufficient (VAS) rats and VAD rats that had not received retinoic acid (RA) and VAD rats that had received RA for 12 or 21 days (d). The CCh-induced increase in bulk modulus was significantly (#, p < 0.05) higher in VAD rats that were untreated or treated for 12 or 21 d with RA, than in VAS rats. In the absence of CCh (solid bars), the bulk modulus was increased in VAD rats that had received RA for 12 or 21 d, compared to VAS rats (+, p < 0.05). **(B) **Comparing the ratio of bulk modulus after carbamylcholine (CCh) to before CCh, at 3 cm PEEP, showed that the CCh-induced increase in bulk modulus was significantly higher in vitamin A deficient (VAD) rats than in VAS rats (*), p < 0.05, n = 9 for each treatment group. 3-way ANOVA, Student-Newman-Keuls post-hoc test.

**Figure 3:**
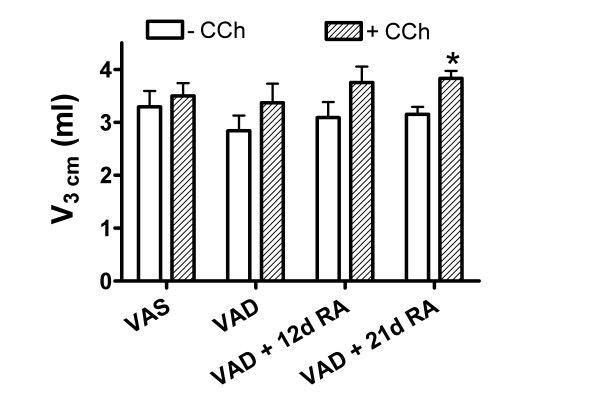
**Volumes of excised lungs at 3 cm H2O did not vary with vitamin A status**. In the absence of carbamycholine (CCh), residual volume (RV) was determined by volume displacement and the volume of air required to maintain 3 cm pressure was ascertained from the deflation pressure volume curve (open bars). A similar determination was made immediately after CCh-administration (hatched bars). Volumes (V, mean ± SEM, n = 8 for each vitamin A treatment group) shown are the sum of RV (volume at 0 cm) and the volume required to maintain 3 cm H2O pressure. (*) V after CCh greater than before CCh (p < 0.05, 2-way ANOVA, Student-Newman-Keuls post-hoc test).

The bulk modulus, measured at 3 cm PEEP, increased after the administration of CCh in both VAS and VAD rats, but the increase was approximately 2-fold greater in VAD rats (Figure 2A). Administration of RA for 12 or 21 days did not ameliorate the heightened CCh-mediated increase in bulk modulus, which remained significantly greater than VAS after both 12 and 21 days RA-treatment. There was a significant increase in the bulk modulus, in the absence of CCh, for VAD lungs that were treated with RA for 12 or 21 days, compared to VAS lungs. In VAD rats, the fold-increase in bulk modulus that was attributable to CCh was greater than the CCh-mediated increase that was observed in VAS rats (Figure 2B). However, VAD rats that received RA showed a smaller increase in bulk modulus after CCh compared to pre-CCh, and the fold-increases in these two groups were not significantly greater than for VAS rats. The static volumes of the lungs were not significantly altered by vitamin A-status and the increase in volume after CCh administration was only significant for VAD rats that received RA for 21 days (Figure 3). The lung volumes at 3 cm H2O did not vary among the various retinoid-treatment groups (Figure 3), so an increase in volume did not significantly contribute to the observed increase in bulk modulus in VAD rats. The volumes (including residual volume) of the lungs that had been inflated to 20 cm H2O also did not vary among retinoid treatment groups. They were 7.0 ± 0.9, 7.1 ± 0.4, and 7.2 ± 0.5 ml (mean ± SEM, n = 4) for VAS, VAD and VAD + 12 d RA, respectively

**Figure 4:**
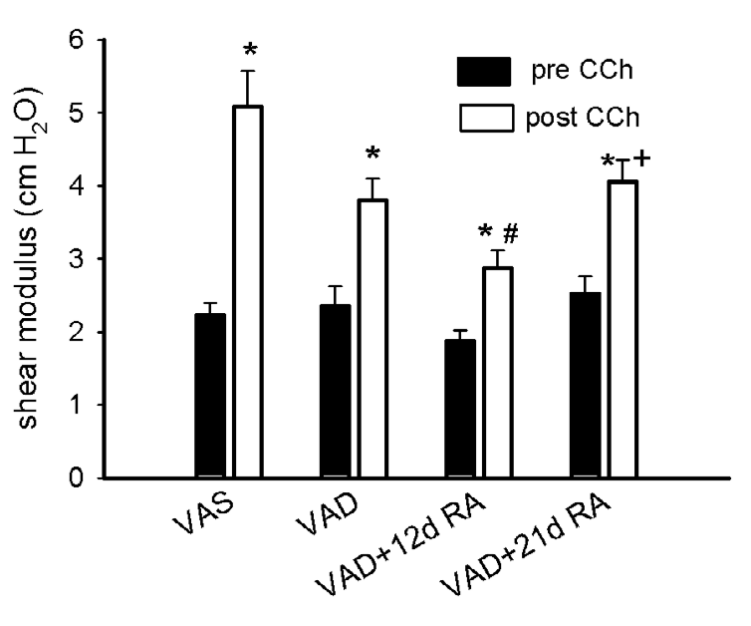
**Shear modulus is decreased in vitamin A deficiency (VAD)**. The shear modulus (mean ± SEM, n = 9 for each treatment group (the same as in Fig. 2) increased significantly after carbamylcholine (CCh), (*) p < 0.05 post-CCh (open bars) compared to pre-CCh (solid bars). (#) p < 0.05, post-CCh for VAD + 12d RA compared to post-CCh for VAS. (+) p < 0.05 VAD + 21 d RA compared to VAD + 12 d RA, post-CCh. 3-way ANOVA, Student-Newman-Keuls post-hoc test.

As expected, the shear modulus increased after the administration of CCh for all categories of retinoid-status. Whereas VAD was associated with a larger increase in bulk modulus after CCh administration, the increase in shear modulus was smaller in VAD than in VAS rats (Figure 4). When measured after CCh administration, the shear modulus of the lungs of VAD rats that had received RA for 12 days was significantly smaller than that observed in VAS rats (Figure 3). In summary, these data indicate that VAD alters the mechanical properties of the lung parenchyma, and the alterations are most evident after CCh-administration. Repletion with RA for 12 or 21 days did not significantly restore the CCh-related changes in bulk modulus, although the bulk modulus in the absence of CCh was affected by RA-administration. After 21 days of RA-administration the shear modulus after CCh returned to a level that was similar to that of VAS rats.

### VAD reduces the concentration of elastic fibers and the quantity of lung elastin

**Figure 5:**
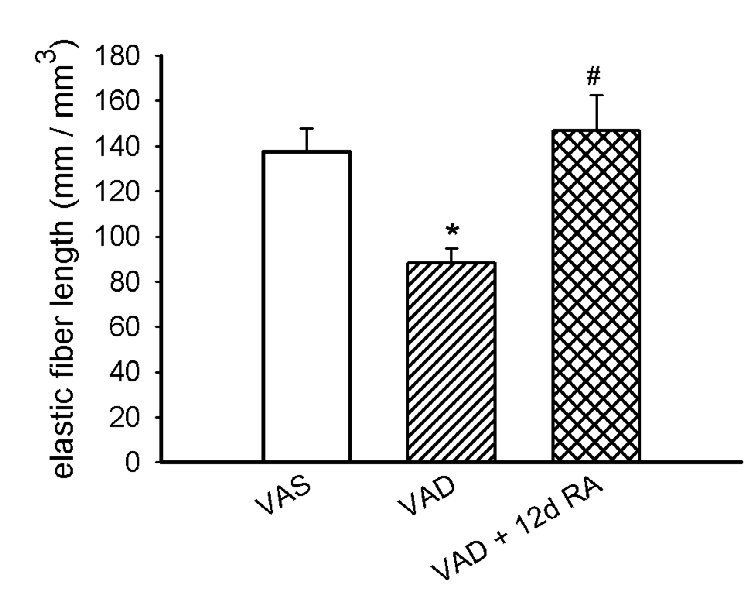
**Elastic fiber concentration (mm length / mm^3 ^parenchyma) was decreased in VAD rats and was restored by retinoic acid (RA)-administration**. The length (mean ± SEM) of elastic fibers per unit volume was decreased in lungs from VAD (n = 9 sections analyzed) rats compared to lungs from VAS rats (n = 11) that were fixed at the same pressure (*) p < 0.05, 1-way ANOVA, Student-Newman-Keuls post-hoc test. The fiber concentration in VAD rats that received RA for 12 days (VAD + 12d RA, n = 6) was significantly greater than for VAD (#), p < 0.05. 3 rats were used for each retinoid-treatment group.

**Figure 6:**
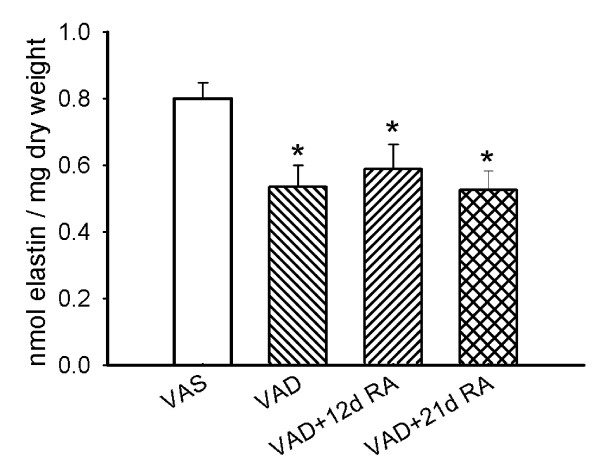
**Lung elastin contents were decreased in vitamin A deficiency (VAD)**. Lung elastin (mean ± SEM, n = 6 for each group), normalized to the dry-defatted lung weight was decreased in VAD compared to vitamin A sufficient (VAS) rats, which was not altered by retinoic acid (RA) treatment for 12 or 21 days. (*) p < 0.05, 1-way ANOVA. Student-Newman-Keuls post-hoc test.

The lungs of some rats from each retinoid-treatment group were fixed at 20 cm H_2_O inflation pressure and were dehydrated and embedded in LR-White resin, using the same methods for all of the lungs. The concentration of elastic fibers, which were detected by an orcein stain, was significantly lower in VAD than in VAS rats and administration of RA for 12 days restored the concentration of elastic fibers (Figure 5). The differences in elastic fiber concentration were not due to differences in the internal surface area. When the fiber concentration (mm fiber length /mm^3 ^of lung) was divided by the internal surface area (mm^2^/mm^3 ^of lung) of the respective lungs, the ratios of fiber length to surface area (mm/mm^2^) were 0.76 ± 0.06 (n = 11), 0.51 ± 0.03 (n = 9, p < 0.01 compared to VAS), and 0.92 ± 0.08 (n = 6, p < 0.01 compared to VAD) for VAS, VAD and VAD + 12d RA, respectively (1-way ANOVA). Elastin, which was resistant to hot alkali treatment, was also reduced in reduced in VAD rats, but unlike the density of elastic fibers that were visualized after orcein-staining, the elastin content was not restored by the administration of RA for 12 days (Figure 6).

### Administration of RA to VAD rats increases tropoelastin mRNA

**Figure 7:**
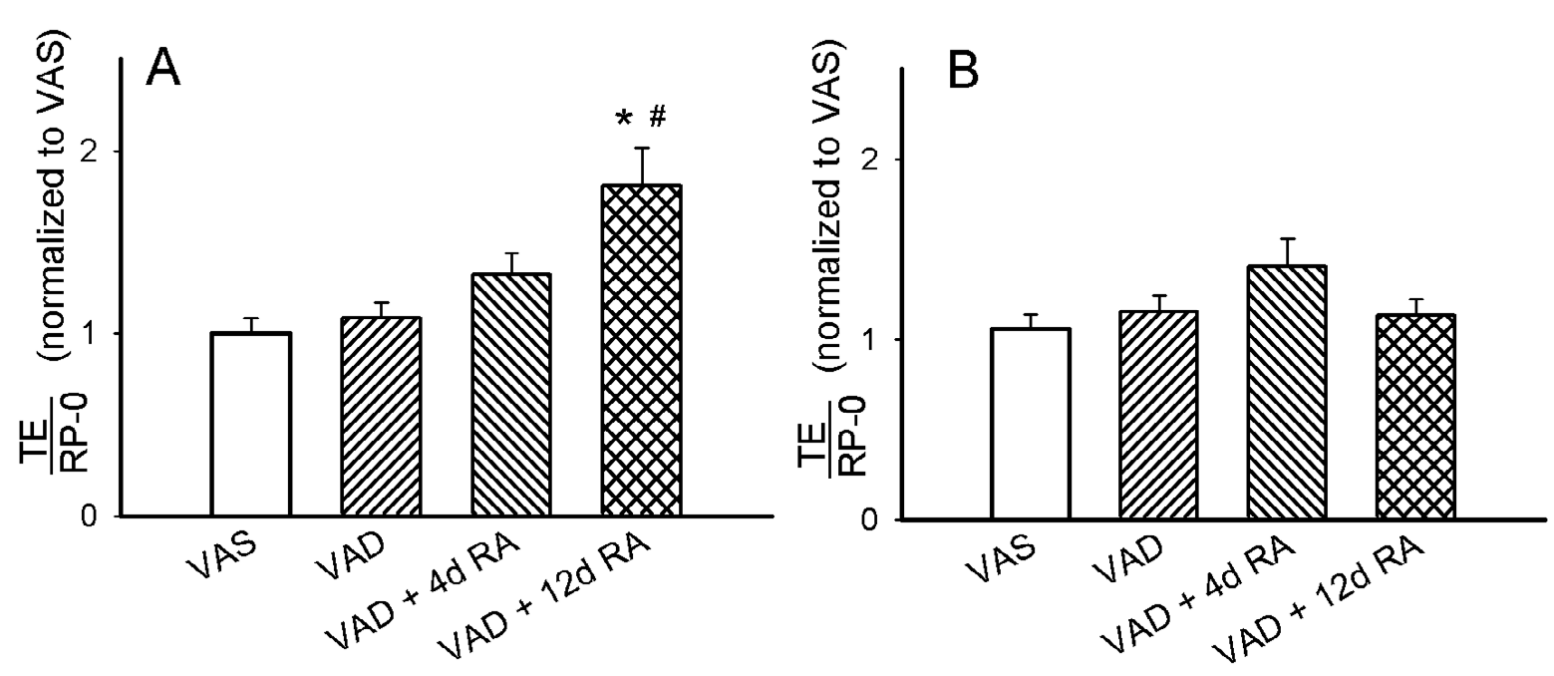
**Tropoelastin (TE) mRNA was increased in the lung parenchyma after retinoic acid (RA) administration**. Lung parenchymal **(A) **and bronchial **(B) **tissues were separated prior to RNA isolation. The filters from Northern analysis were re-probed for the constitutively expressed mRNA for ribosomal phosphoprotein P-0 (RP-0) to correct for differences in the amounts of RNA loaded. The density of TE mRNA was expressed relative to that for RP-0 for each lane, and normalized to the mean density for RNA from VAS rats within each Northern analysis. Data are mean ± SEM, n = 9 rats for each retinoid-treatment condition. Treatment with RA for 12 days (VAD + 12d RA) increased lung but not bronchial TE mRNA (*) p < 0.05, 2-way ANOVA. Treatment with RA for 4 days (VAD + 4d RA) did not significantly increase lung or bronchial TE mRNA.

Because administering RA for 12 days increased the concentration of elastic fibers in VAD rats, we investigated the steady state-level of tropoelastin (TE) mRNA in lung and bronchial tissues that were isolated from VAS rats and VAD rats that were untreated or had received RA for 4 or 12 days. Northern analyses were preformed and the densities of the bands for tropoelastin were normalized to ribosomal phosphoprotein P-0 (RP-0), to account for inadvertent differences in the quantities of RNA that were loaded in various lanes. The results for lung and bronchial RNA shown in Figures 7A and 7B, respectively demonstrated that 12 days of RA-administration significantly increased TE mRNA in lung tissue, but not bronchial tissue. There was a trend towards an increase in TE mRNA in bronchial tissue after 4 days of RA administration (p = 0.1).

### VAD is associated with an increase in static lung elastance

**Figure 8:**
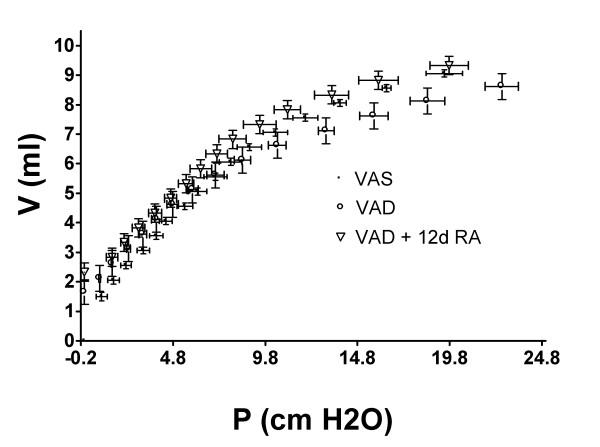
**Deflation pressure-volume analysis in the absence of carbamylcholine**. Deflation pressure (P)-volume (V) curves are shown for four rats from each vitamin-A treatment group (mean ± SEM; VAS, vitamin A sufficient; VAD, vitamin A deficient; VAD + 12d RA, VAD treated for 12 days with RA).

**Figure 9:**
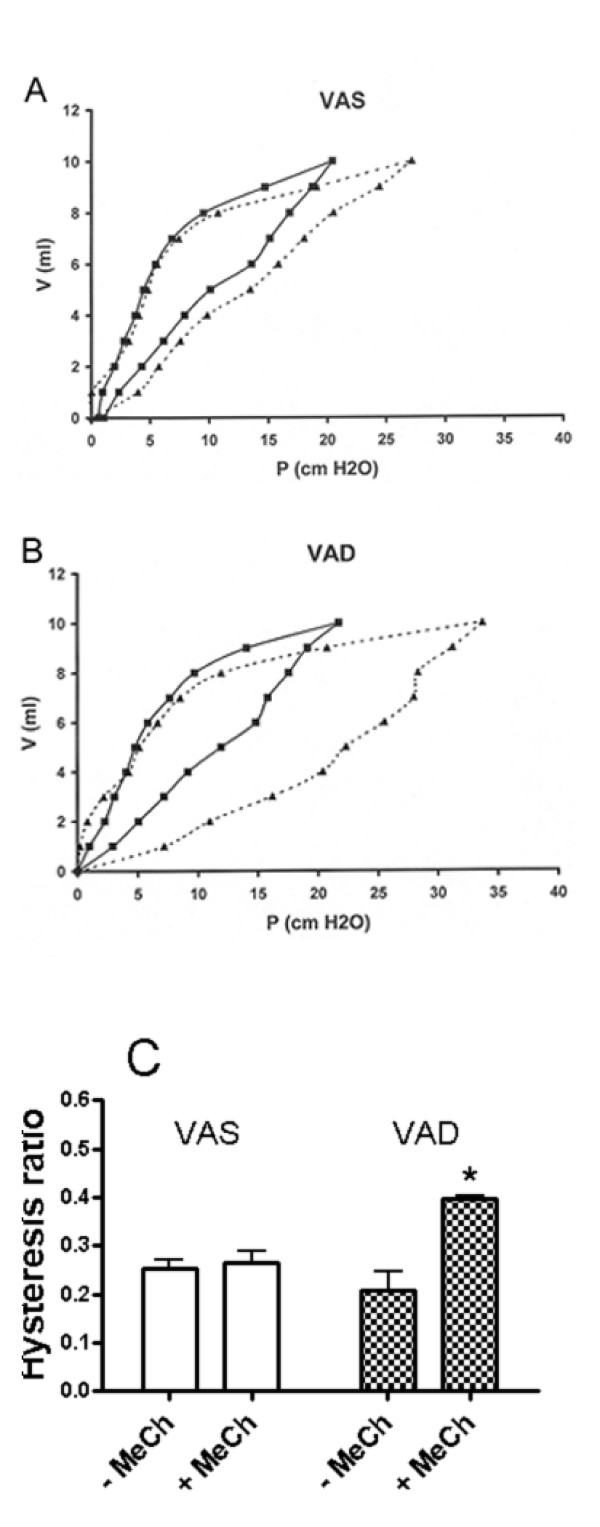
**Cholinergic administration produces a larger increase in hysteresis in VAD rats**. The effects of cholinergic administration on pressure-volume hysteresis are shown for one representative VAS (**A**) and one representative VAD (**B**) rat. Solid line, prior to methacholine administration; broken line, immediately following methacholine administration. The mean ± SEM of the hysteresis ratio (**C**) was calculated for three VAS (open bars) and 3 VAD (checked bars) rats both before, and was significantly increased in VAD rats (p < 0.01, 1-way ANOVA, Student-Newman-Keuls post-hoc test) by cholinergic administration.

VAD significantly increased the slope of the deflation pressure-volume curve and this was not restored by the administration of RA for 12 days (Figure 8). The slopes (ΔP/ΔV) were 1.136 ± 0014 (4), 1.297 ± 0.014 (4)*, and 1.241 ± 0.025 (4) for VAS, VAD and VAD + 12d RA, respectively. (*, VAS versus VAD) p < 0.05, 1-way ANOVA, Student-Newman-Keuls post hoc test. The effects of CCh-administration on the pressure-volume hysteresis for a representative VAS and VAD rat are shown in Figure 9A and 9B, respectively. In VAD rats methacholine-administration strikingly increased the pressure that was required to inflate the lungs compared to the effects of methacholine on VAS lungs. This rightward shift in the inflation portion of the pressure-volume curve contributed to a large CCh-mediated increase in the hysteresis of VAD (Figure 9B) compared to VAS lung (Figure 9A). This was a consistent finding in two other VAS and VAD rats, as indicated by the significant increase in the hysteresis ratio (mean ± SEM, n = 3), shown in Figure 9C.

### The airway contraction index was decreased in VAD rats

**Figure 10:**
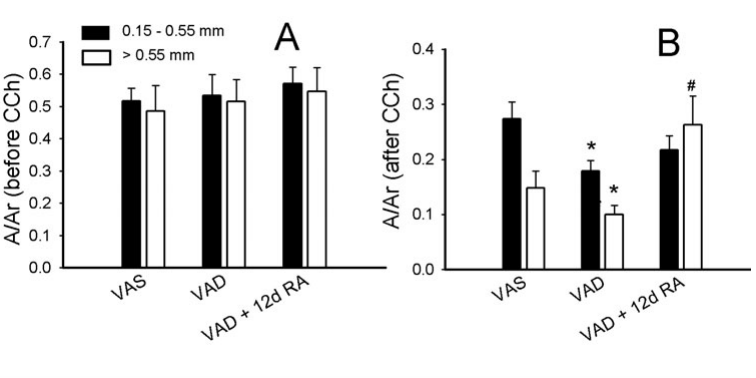
**Airway contraction index is decreased in vitamin A deficient (VAD) rats**. Airway contraction index (A/Ar, mean ± SEM, n = 9 rats for each retinoid treatment group). Solid bars are bronchi with diameters from 0.15 to 0.55 mm; open bars are bronchi with diameters greater than 0.55 mm. (**A**) shows that the contraction index prior to CCh-administration did not vary among the various retinoid treatment groups. After CCh-administration (**B**) the A/Ar was lower in VAD airways and was restored by retinoic acid (RA). (*) p < 0.05, VAD versus vitamin A sufficient (VAS). (#) p < 0.05, VAD + 12 days of RA versus VAD. Comparisons were between bronchi within the same range of diameters.

The airway contraction index is a morphometric assessment of reduction in airway caliber and compares the actual area internal to the epithelial basement membrane to the idealized maximal area if the bronchus was completely dilated. Therefore a smaller contraction index (A/Ar) correlates with a greater degree of luminal narrowing. Figure 10A shows the contraction index did not vary among the various retinoid treatment groups for bronchi that that had not been exposed to CCh. Figure 10B shows the contraction index for bronchi in lungs after exposure to CCh. Airways were stratified according to their diameter because the degree of contraction is dependent on the initial diameter, as well as the response to the cholinergic agent [30]. After CCh-administration, the index was significantly lower in VAD rats at both ranges of diameter than in VAS rats (Figure 10B). After 12 days of exposure to RA, the contraction index increased and was significantly higher than in untreated-VAD rats, for airways of diameter greater than 0.55 mm.

### VAD accentuates the distortion of the gas-exchange region in VAD rats

**Figure 11:**
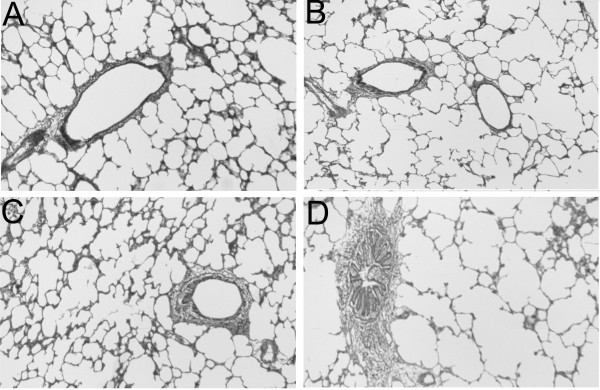
**Airway contraction and airspace distortion after carbamylcholine (CCh) administration**. Lungs from vitamin A sufficient (VAS), A and C, or from vitamin A deficient (VAD), B and D, were fixed while inflated at 10 cm H_2_O pressure without, A and B, or with prior exposure to CCh, C and D. Alveoli and alveolar ducts were more dilated in VAD lungs particularly after CCh. Panel D also shows atelectatic areas which accompanied areas of hyperinflation in VAD lungs.

Morphometric analysis of lungs from VAS and VAD rats, which had been inflated to 10 cm H_2_O pressure, without or immediately after exposure to CCh was performed to assess hyperinflation and atelectasis in the region of the alveoli and alveolar ducts. Representative photomicrographs of VAS and VAD lung are shown in Figure 11, which illustrates that VAD lungs (panels B and D) have more enlarged airspaces than VAS lungs (panels A and C) and that the enlargement is more pronounced after CCh administration. The results shown in Table 1 indicate that whereas the Lm was similar in CCh-unexposed VAS and VAD rats, CCh administration led to more pronounced airspace enlargement in VAD rats. This was evidenced as a larger Lm and SD Lm in VAD rats, indicating that the alveolar ducts and alveoli were more dilated with air and that the dilation was more heterogeneously distributed in the lungs of VAD rats. Whereas the percentage of alveolar and alveolar duct tissue (ATI), as opposed to air, was similar in VAS and VAD lungs after CCh-administration, there was more heterogeneity in the tissue density among different portions of the lungs of VAD rats (greater SD ATI).

**Table 1:** Morphometric analysis of the gas-exchange region from carbamylcholine (CCh) exposed and unexposed rats

**CCh (mg/ml)**	**vitamin A status**	**Lm**	**mean SD Lm**	**ATI**	**mean SD ATI**
0	VAS	40.15 ± 3.34 (n = 3)	4.89	20.49 ± 2.50 (n = 3)	2.78
0	VAD	40.74 ± 2.72 (n = 3)	3.23	16.69 ± 0.65 (n = 3)	1.63
12	VAS	36.10 ± 4.07 (n = 4)	5.88	20.41 ± 1.92 (n = 4)	2.81
12	VAD	44.41 ± 4.92* (n = 4)	9.84*	20.04 ± 1.12*† (n = 4)	4.15*†

Rats were exposed to carbamylcholine (CCh) or remained unexposed. Lm and ATI are expressed as mean ± SEM (n=number of rats used). Mean SD Lm was the mean of the standard deviations (SD) of the mean chord lengths (Lm) for the 9 sections, which were randomly photographed for the left lung of each rat. n = 4 lungs from CCh-exposed VAS and VAD rats. n = 3 lungs from unexposed VAS and VAD rats. The atelectasis index (ATI) was the percent of pixels that represent tissue (as opposed to air). The SD ATI was calculated from the SD of the ATI for the same 9 sections from each lung that were used to determine the Lm. (*) p < 0.05, VAS vs. VAD within CCh-treated group. (†) p < 0.05 VAD comparing 0 to 12 mg/ml CCh.

## Discussion

Our previous studies of rat lungs *in vivo *have shown that the cholinergic-induced increase in total pulmonary elastance (which in this preparation is influenced by both the lung and the chest wall) is greater in VAD rats, and that RA-treatment restores the increase in elastance to a level, which is similar to that observed in VAS rats [1]. Elastance increases as tissue stiffness increases. In the lung, elastance is increased (a) when lung volumes approach total lung capacity, (b) by atelectasis, or (c) by an increase in rigid structural components (such as collagen) or (d) by an increase in hysteresis, which could result from alterations in alveolar surface tension or disruption of the elastic fibers [32,33]. In order to more specifically examine the contribution of the lung parenchyma to the exaggerated CCh-mediated increase in total pulmonary elastance that was observed VAD rats, we ventilated the lung *ex vivo *at a small tidal volume. This approach eliminated the contributions of the chest wall and of innervation and avoided the confounding effects of air-trapping that can be induced by large-volume oscillations. We found that the CCh-mediated increase in the elastic bulk modulus was exaggerated in VAD rats. This was manifest as an increase in the pressures required to expand the lung during inflation and a significant increase in hysteresis. In contrast, the CCh-mediated increase in shear modulus was diminished in VAD rats. Administration of RA for up to 21 days did not significantly reverse the effects of vitamin A deficiency on the bulk modulus, but there was a partial normalization of the shear modulus after 21 days of RA-treatment. The VAD-related alterations in the mechanical properties of the lung parenchyma were accompanied by a decrease in the concentration of parenchymal elastic fibers and in lung elastin. The administration of RA for 12 days increased TE mRNA but did not restore the 0.1 M NaOH-resistant lung elastin, although the concentration of parenchymal elastic fibers was increased. Therefore, decreases in lung parenchymal elastic fibers and total pulmonary elastin likely contribute to but do not completely account for to the exaggerated CCh-mediated increase in the elastance and bulk modulus in VAD rats.

Others have shown, using a qualitative pathologic grading system in rats, that VAD is associated with patchy atelectasis as well as emphysema [33]. Our previous morphometric study, using lungs that were inflated to 20 cm H_2_O confirmed the presence of emphysematous areas [1]. Terminal airway closure that occurs after the administration of aerosolized cholinergic agents results in a non-uniform distribution of atelectatic and hyperexpanded areas of parenchyma, which could exaggerate the pre-existing abnormalities that are associated with VAD [14]. The data in Table 1 are consistent with this statement, and show that both the SD Lm and SD ATI are increased in VAD relative to VAS lungs, following CCh administration. The exaggerated CCh-mediated increase that we observed in the bulk modulus reflects an increase in the elastance of the lung parenchyma of VAD rats. The deflation volume-pressure characteristics of the excised lung are also consistent with an increase in lung elastance in VAD. This differs from what one would expect in a uniformly emphysematous lung for which elastance would decrease. Furthermore, one might expect that the decrease in elastic fiber concentration (length per mm^3 ^lung parenchyma) and lung elastin that we observed in VAD rats would be accompanied by a decrease in lung elastance. Therefore, another anatomical abnormality must contribute to the exaggerated increase in parenchymal lung elastance after CCh-administration. It is likely that this abnormality involves localized areas of atelectasis and hyperinflation, which are exaggerated by CCh-administration (see Figure 11 and Table 1). From Figure 9 it is clear that higher pressures are required to expand VAD lungs, compared to VAS lungs, after cholinergic administration. This is particularly obvious at low lung volumes that are similar to those which were used to ventilate the excised lungs during the measurement of the bulk and shear moduli. An increase in surface tension in atelectatic regions is probably the major contributor to this increase in elastance and therefore the bulk modulus. These increased inflationary pressures in cholinergic-exposed VAD lungs resulted in an increase in the hysteresis of VAD compared to cholinergic-exposed VAS lungs (Figure 9).

The VAD-induced suppression of the CCh-mediated increase in the shear modulus requires an alternate explanation (Figure 4). Although the shear modulus increased as expected after CCh-administration in VAD lungs, the increase was less than in VAS lungs. The shear modulus reflects the ability of the lung parenchyma to resist distortion. As the lung is progressively inflated, the "struts" which surround the airspaces become more distended and rigid [12]. This leads to a greater resistance to a distorting shear stress. CCh administration increases alveolar distortion resulting in hyperexpanded alveoli, which stiffens the lung and increases the shear modulus [13]. The cholinergic-induced hyperexpansion and stiffening of the struts appears to occur more uniformly (SD Lm is lower) in VAS lungs, which are not restricted by pre-existing distortion from atelectasis and airspace enlargement, than in VAD lungs. In the areas of VAD lung where alveolar hyperexpansion occurs, there is less elastic tissue to resist shape distortion, which would result in a lower shear modulus. The decrease in elastic tissue likely contributes to the smaller CCh-induced increase in the shear modulus of VAD. The blunted CCh-induced increase in shear modulus in VAD rats likely contributes to their airway hyperresponsiveness, because the shear modulus is thought to be the most important characteristic that mediates airway-parenchymal interdependent opposition to airway contraction [34].

We observed that VAD, which occurs after the major peak of pulmonary elastin synthesis has occurred, is accompanied by a decrease in lung parenchymal elastin. The loss of elastin in VAD was manifest as a decrease in the alveolar septal elastic fiber concentration (mm length per mm^3 ^lung parenchyma) and in the quantity of elastin that was resistant to digestion in the presence of 0.1 M NaOH at 98° (Figures 5 and 6, respectively). We also made the novel observation that RA stimulates elastin synthesis and the deposition of elastic fibers, which are important determinants of the mechanical properties of the parenchyma. Northern analysis demonstrated that 12 days of RA-administration increased the steady-state level of tropoelastin mRNA in the lung parenchyma, which is consistent with the restoration of elastic fiber concentration after 12 days of RA-treatment (Figure 7). However, we did not observe an increase in alkali-resistant elastin after 12 days of RA-administration. This may result from one or more of several factors. First, orcein can stain "immature" elastic fibers that contain a larger proportion of microfibrils than thicker fully cross-linked "mature" elastic fibers [35]. Only the "mature" elastic fibers are resistant to the alkali treatment, which underestimates that quantity of newly formed, incompletely cross-linked elastin. Secondly, our morphometric analysis of elastic fiber concentration did not account for the thickness of the fibers, only the length per unit volume of lung. Therefore, thin newly formed elastic fibers would contain less elastin that could be detected by our biochemical analysis, but the fibers would be detected by our method for determining the concentration of elastic fibers, which is only dependent on the length of the fiber network and not on its thickness.

The airway contraction index after CCh-administration was lower in VAD rats relative to VAS controls. Administration of RA for 12 days was associated with a restoration of the contraction index (after CCh) of airways >0.55 mm diameter to a level that was similar to VAS rats. These data are consistent with our previous study, which demonstrated that 12 days of RA administration restored total (lung plus chest wall) pulmonary elastance and resistance [1]. The data for the contraction index should be considered in light of our observation that RA-administration does not reverse the exaggerated CCh-mediated alterations in bulk and shear moduli in VAD rats. This consideration suggests that the RA-mediated correction of the increase in total pulmonary elastance that we previously observed in VAD rats with intact chest walls was primarily due to factors within the airways or chest wall rather than the lung parenchyma [1]. If RA had corrected the lung parenchymal factors, then we would have expected to observe a correction in the VAD-related abnormalities in the parenchymal elastic modulus. These findings suggest that although VAD alters the elastic fiber system, alveolar architecture, and mechanical properties of the lung parenchyma; treatment with RA for 12 days corrects a VAD-related abnormality in the airway, rather than the parenchyma. When considered along with our prior observation that 12 days of RA treatment normalizes the expression of the muscarinic receptor-2, these findings suggest that the salutary effect of administering RA for 12 days is limited to the airways [1].

## Conclusion

Multiple factors contribute to airway hyperreactivity in vitamin A deficiency including changes in the architecture of the alveoli and alveolar ducts. Aerosolization of cholinergic agents results in more distortion and heterogeneity of airspace size, and in particular atelectasis, that may contribute to the exaggerated CCh-mediated increase in bulk modulus in vitamin A deficient rats. There is also a decrease in elastin and the concentration of elastic fibers in vitamin A deficiency, which may reduce the ability of the parenchyma to resist deformation after CCh administration, resulting in a smaller CCh-mediated increase in the shear modulus than in vitamin A sufficient rats. Retinoic acid administration restores the parenchymal elastic fibers but does not restore the CCh-induced responses of the bulk and shear moduli to the pattern that was observed in VAS rats. Therefore, architectural changes that are not directly related to elastin also influence airway-parenchymal interdependence and enhance airway hyperreactivity.

## Abbreviations

**ATI**, atelectasis index; **CCh**, carbamylcholine; **E**, elastance; **HPLC**, high performance liquid chromatography; ***k***, bulk modulus; **L**_m_, mean chord length; **PBS**, 15 ml of 137 mM NaCl, 8 mM Na_2_HPO_4_, 2.7 mM KCl, 1.5 mM KH_2_PO_4_, pH 7.4; **PEEP**, positive end-expiratory pressure; **R**, resistance; **RA **retinoic acid; **TE**, tropoelastin; **VAD**, vitamin A deficient or vitamin A deficiency; **VAS**, vitamin A sufficient; μ, shear modulus.

## Competing interests

The three authors declare that neither has a completing interest that would influence the objectivity of these findings.

## Grant Support

The authors thank the Veterans Affairs Research Service (Merit Review Grant) National Heart, Lung and Blood Institute (HL53430, HL62861) for supporting this research.

## Authors' contributions

SEM planned the experiments, performed the physiological measurements, wrote the manuscript, and performed some of the morphometry. AJT performed the studies of elastic fiber concentration, lung elastin contents, airway contraction index and assisted with the preparation of the manuscript. AJH performed the Northern analyses and made substantive contributions to the writing of the manuscript.
